# Children With Autism Spectrum Disorder With Regression Exhibit a Different Profile in Plasma Cytokines and Adhesion Molecules Compared to Children Without Such Regression

**DOI:** 10.3389/fped.2018.00264

**Published:** 2018-09-26

**Authors:** Antonio Gomez-Fernandez, Maria J. de la Torre-Aguilar, Mercedes Gil-Campos, Katherine Flores-Rojas, Maria D. Cruz-Rico, Pilar Martin-Borreguero, Juan Luis Perez-Navero

**Affiliations:** ^1^Department of Pediatrics, Reina Sofia University Hospital, University of Córdoba, Maimónides Institute for Biomedical Research of Córdoba (IMIBIC), Cordoba, Spain; ^2^Pediatric Metabolism Unit, Reina Sofia University Hospital, Maimónides Institute for Biomedical Research of Córdoba (IMIBIC), University of Córdoba, Centro de Investigación Biomédica en Red-Fisiopatología de la Obesidad y Nutrición (CIBEROBN), Cordoba, Spain; ^3^Department of Biochemistry and Molecular Biology II, Institute of Nutrition and Food Technology, Center of Biomedical Research, University of Granada, Granada, Spain; ^4^Department of Child and Adolescent Clinical Psychiatry and Psychology, Reina Sofia University Hospital, Maimónides Institute for Biomedical Research of Córdoba (IMIBIC), Cordoba, Spain

**Keywords:** autism spectrum disorder, cell adhesion molecules, children, cytokines, neurodevelopmental regression

## Abstract

**Background:** In the etiopathogenesis of autism spectrum disorder (ASD), it has been suggested that a proinflammatory condition, as well as an alteration in adhesion molecules in the early stages of neurodevelopment, may play a role in the pathophysiology of the disorder. This study set out to evaluate the plasma levels of certain inflammatory cytokines, adhesion molecules, and growth factors in a sample of pediatric patients with ASD and compare them to the levels in a control group of healthy children.

**Methods:** Fifty-four children (45 males and nine females) aged 2-6, who were diagnosed with ASD, and a control group of 54 typically-developing children of similar ages were selected. The diagnosis of ASD was carried out in accordance with the DSM-5 criteria and the data obtained from a developmental semi-structured clinical interview and the ADOS evaluation test. Additional testing was carried out to identify the children's developmental level and severity of ASD symptomatology. Patients with ASD were further divided into two subgroups based on developmental parameters: ASD children with neurodevelopmental regression (AMR) and ASD children without neurodevelopmental regression (ANMR). Analyses of plasma molecules, such as cathepsin, IL1β, IL6, IL8, MPO, RANTES, MCP, BDNF, PAI NCAM, sICAM, sVCAM and NGF, were performed.

**Results:** Higher levels of NGF were observed in the ASD group compared with the levels in the control group (*p* < 0.05). However, in the analysis of the ASD subgroups, lower plasma levels of NCAM and higher levels of NGF were found in the group of ASD children without developmental regression compared to the levels in the group of typically-developing children.

**Conclusions:** These results suggest differences that could be related to different pathophysiological mechanisms in ASD. There is not a specific profile for the expression of relevant plasma cytokines, adhesion molecules or growth factors in children with ASD compared with that in typically-developing children. However, in the ANMR and AMR subgroups, some of the adhesion molecules and neuronal growth factors show differences that may be related to synaptogenesis.

## Introduction

Autism spectrum disorder (ASD) is a neurodevelopmental disorder that is characterized by persistent deficits in social communication and social interaction across multiple contexts and by the presence of repetitive and restricted patterns of behaviors, activities, and interests. These deficits are present from early childhood, although some difficulties may not manifest themselves until the demands of the environment exceed the capacity of the child (1). ASD mainly affects males, by a ratio of 4:1 (2), and is generally detected in the second year of life.

It is well established that children with ASD do not constitute a homogeneous clinical group, and many different pathologies show a similar constellation of behavioral symptoms that converge within ASD. The existence of developmental regression in some children with ASD has been corroborated by multiple studies (3). Children with ASD who lose skills belong to a subgroup designated with the term “regressive autism.” Regressive autism usually refers to a child whose parents report an early history of normal development for 12–24 months, followed by a loss of previously acquired skills. Language regression is the most obvious form of regression, but regressive autism can also be accompanied by more global regression involving loss of social attention, social skills and social interest (4).

Knowledge about the etiology and neuropathology of ASD remains elusive however, and no biological markers have been found. Most studies concur that there has been a significant increase in the prevalence of autism, affecting approximately 0.75–1.55% of the pediatric population (5, 6). It has recently been reported that the prevalence of ASD reaches nearly 3% in some communities in the United States, representing an increase of 150% since 2000 (7).

Various studies published in recent years have supported the idea that a proinflammatory situation may play a role in the pathophysiology of ASD (8). An increase in proinflammatory cytokines has been reported in samples of post-mortem brain tissue and cerebrospinal fluid, plasma, and mononuclear cell cultures (9, 10). Levels of tumor necrosis factor alpha (TNF-α), interleukins 6 and 8 (IL-6, IL-8), monocyte-macrophage colony stimulating factor (GM-CSF) and interferon gamma (IFN-γ) have also been reported as being higher in the brains of patients with ASD than in the brains of the healthy population (11). The increase in cytokines may indicate an impaired immune response, with a predominant response of Th2 cells. Some authors have suggested the existence of an endophenotype of ASD linked to an autoimmune dysregulation (12). Others (13, 14) have described altered levels of immune mediators linked to greater deterioration in behavior. It has thus been suggested that a dysregulation of the immune response may be related to the behavior and cognitive impairment of children with ASD.

Chemokines and their receptors have been implicated in the circulation and movement of mononuclear cells in the CNS, mediating the recruitment of myeloid cells into areas of damage and inflammation. Increased levels of various chemokines (macrophage inflammatory proteins [MIP-1B], regulated on activation, normal T cell expressed and secreted [RANTES], monocyte Chemoattractant protein-1 [MCP-1]) have been reported in astrocytes in the cerebellum and brain tissue, as well as in peripheral blood in children with ASD. They have been linked to a deterioration in communication skills, stereotyped behaviors and hyperactivity, and an impairment of cognitive functions and adaptive skills (15, 16). The cell adhesion molecule is an Ig superfamily adhesion molecule (CAM) with an established role in axon growth and repulsion. Serum samples have been reported to exhibit a decrease in the levels of vascular and intercellular cell adhesion molecules, notably sPECAM-1 (soluble platelet-endothelial cell adhesion molecule-1) and sVCAM-1 (soluble vascular cell adhesion molecule-1), which can also play a role in the development of ASD (17).

Multiple hypotheses have been put forward to account for the etiopathogenesis and developmental trajectories of ASD. The roles of cytokines, adhesion molecules and growth factors have not been clarified precisely in this regard, but they may have an adverse bearing on the establishment of neuronal synapses and their functioning in a situation of greater synaptic plasticity. The goal of the present study was therefore to analyze the plasma levels of some relevant molecules in children with ASD and to compare these to the values exhibited by a group of typically-developing children. The study also focused on the possible relationships between the levels of such cytokines and the clinical severity of ASD core symptomatology and the regression phenotype in ASD.

## Materials and methods

This observational case-control study was approved by the Clinical Research and Bioethics Committee at the authors' hospital and was conducted in full compliance with the fundamental principles established in the Declaration of Helsinki. The data relating to the ASD patients were collected at the time they were recruited into the study. The selected subjects were incorporated into the study after all the criteria for inclusion were fulfilled, and informed written consent was obtained from the children's legal guardians.

### Subjects

The patients with ASD were recruited from the Department of Child and Adolescent Clinical Psychiatry and Psychology at the authors' hospital. Some of the patients (aged 2–3) were recruited at the time of their ASD diagnosis, whereas the older patients (aged 3–6) were selected from among those who had already been diagnosed at the same unit. The control group comprised children diagnosed with phimosis and hernias, lacking any other pathology, who attended preoperative anesthesia consultations prior to surgical corrections. None of the controls belonged to the same families as the ASD cases.

The diagnosis of ASD was based on the clinical judgment of professionals specialized in the identification of the unique developmental profile of subjects with ASD. The diagnostic decision was based on the information obtained from semi-structured clinical development interviews and psychological and behavioral tests internationally recognized as reliable and valid for this purpose. In this study, two clinical psychologists, a psychiatrist and an occupational therapist with extensive clinical experience and training in diagnostic tests for research in ASD performed the diagnosis using the criteria established by DSM-5 (1). Two specialized pediatricians reviewed the medical histories of the children and performed examinations of all the children.

Inclusion criteria: the ASD children were 2–6 years old with a clinical diagnosis confirmed by validated scores. Additionally, a control group of typically-developing children was selected and matched to the ASD group by gender and age. A physical examination, assessing neurological development and a blood analysis were performed to confirm that they were healthy children.

Any children with ASD presenting other neurological, metabolic or genetic diagnoses were excluded, as were children receiving medical treatment for autism-related behavioral comorbidities. The group of ASD children was further divided into two subgroups based on the presence or absence of neurodevelopmental regression during the first two years of life, which was assessed using a five-item questionnaire following the guidelines used by the Autism Diagnostic Interview-Revised (ADI-R) for the evaluation of this process (18). The ASD children who obtained a score equal to or >3 were included in the neurodevelopmental regression ASD group (AMR), and those with a score of less than 3 were included in the non-neurodevelopmental regression ASD group (ANMR). The ASD group was also classified according to whether children presented neurodevelopmental delay (i.e., a score lower than 70 in the cognitive quotient of the Battelle developmental test).

### Standardized diagnostic measurements and assessments of ASD severity

All the children with ASD underwent an initial developmental clinical interview, which identified the core symptomatology of ASD according to DSM-5 clinical diagnostic criteria. Additionally, the following tests were administered to all the children:

- ***Autism Diagnostic Observation Schedule-2*** *(ADOS-2)* (19). This is a standardized and semi-structured assessment scale designed to measure communication, social interaction, play, and the imaginative use of materials. The children were given an ADOS module consistent with their language development and age. This was administered by two clinical psychologists with official formal training in the administration and quantitative interpretation of the test for research purposes. All children with ASD in the study exceeded the cut-off point for the diagnosis of ASD.
- ***Pervasive Developmental Disorders Behavior Inventory Parent Ratings*** (PDDBI), standardized version for the Spanish-speaking European population. (20) This test was used to evaluate the symptomatic severity of pervasive developmental disorders in ASD patients aged from 2 to 6 years. This test, which was administered to all the parents (not teachers) of the children with ASD, evaluates the characteristic ASD core behavioral deficit (deficits in social interaction, language and pragmatic communication and stereotyped behavior), additional behavioral difficulties (fears, aggressive behaviors), and adaptive behaviors (scales of the children's social, linguistic, and learning skills). All the children with ASD in the study obtained a score ≥30.
- ***Childhood Autism Rating Scale Test (CARS-2****)* (21). This scale was used for the quantification of the severity of autism pathology: mild, moderate or severe.
- ***Battelle Developmental Inventory, Second Edition (BDI-2)*** (22). This test assesses the child's current level of development and functioning in five areas (i.e., personal/social, adaptive, motor, communication, and cognitive areas).
- ***Strengths and Difficulties Questionnaire (SDQ)*** (23): Two to four years. This questionnaire was used to assess the presence of behavioral difficulties, as well as adaptive behaviors.

### Clinical evaluation

A thorough developmental clinical history, including major childhood illnesses and immunisations, was taken. A physical examination was also conducted with a special emphasis on neurological and nutritional status. Anthropometric measurements (weight, height and body mass index) were obtained using standard techniques, and validated nutritional questionnaires were also administered.

### Biochemical analysis

After overnight fasting, blood samples were collected from the children with ASD and the healthy controls from the antecubital vein into 6-ml blood collection tubes containing EDTA. After centrifugation at 3,500 g for 10 min, plasma was divided into aliquots and processed within 2 h from sampling, and then frozen at −80°C until analysis. Blood count and a general biochemical analysis were performed to confirm the absence of other diseases. C-reactive protein (C-CRP) (mg/l) was also measured as an inflammatory marker. These analyses were performed in the hospital laboratory using colorimetric, enzymatic, kinetic, indirect potentiometry, or immunoturbidimetric methods previously standardized, using an automatic autoanalyzer (Roche-Hitachi Modular PYD autoanalyzer, Roch Laboratory Systems, Mannheim, Germany).

In the light of observations made in the existing literature (24, 25), molecules that have previously been linked to autism were selected for plasma analysis: IL-1beta, IL-6 and IL-8, MCP-1, TNF-α, myeloperoxidase (MPO), RANTES, cathepsin D, brain-derived neurotrophic factor (BDNF), hepatocyte growth factor (HGF), nerve growth factor (NGF), plasminogen activator factor (PAI) and cell adhesion molecule (NCAM, sICAM, VCAM).

These biomarkers were analyzed through simultaneous detection of multi-analytes using LINCOplex assay kits and Luminex xMAP Detection Technology. The sensitivity of this assay enabled the detection of chemokine concentrations to the following concentrations: IL1-β, 0.4183 pg/ml; IL-6, 0.2 pg/ml; IL-8, 0.3 pg/ml; MCP-1, 2 pg/ml; TNF-α, 0.3 pg/ml; MPO, 20 ug/L; cathepsin D, 8.06 ug/L; RANTES: 12 ug/L; BDNF, 0.23 ug/L; HGF, 4 pg/ml; NGF, 0.3 pg/ml; plasminogen activator factor (PAI), 0.48 ug/L; NCAM, 4.81 ug/L; sICAM-1, 6.29 ug/L, and sVCAM, 6.44 ug/L).

### Statistical analysis

With regard to the size of the ASD sample used in this study, given that the prevalence of ASD in Spain is calculated at 1% (5), 50 patients with ASD needed to be recruited for the study, taking into account a 5% margin of errors and a 95% confidence interval. The sample size for this study was calculated based on the most relevant inflammatory parameters using published data results (16), choosing the one that required a larger sample size (RANTES). Accepting alpha errors of 0.05 and beta errors of 0.2 in a bilateral contrast, 54 subjects per group were required to detect a difference equal to or >400 pg/ml. The standard deviation was assumed to be 720.

The values of the molecules that were not detected were considered to be lost values, and the statistical study was performed on the remaining values. The data are expressed as mean ± SD (95% confidence intervals), median (IQR) or absolute (relative frequencies). The Shapiro–Wilk test was used for typically distributed data. Homogeneity of variances was estimated using Levene's test. The mean values for typically-distributed continuous variables among groups were compared using the unpaired Student's *t*-test. The Mann–Whitney *U*-test was used for asymmetrically distributed data. Bonferroni correction was used for multiple comparisons to avoid chance. Categorical variables were assessed using the χ2-test or the Fisher exact test.

Comparisons between subgroups of ASD and with the control group were performed using ANOVA, with the Sidak correction for post hoc comparisons. Correlations between the biomarker levels and the scores resulting from the various tests carried out were performed using Spearman's p (rho). All the tests were two-tailed, and a *P*-value < 0.05 was considered to be statistically significant. The data were analyzed using the Statistical Package for the Social Sciences (SPSS for Windows, release 18.0.0 2010, SPSS Inc, Chicago, IL, USA).

## Results

Initially, 57 ASD children were selected for this study. Of these, three children were later excluded for not meeting the diagnostic criteria for ASD during the subsequent follow-up interviews at 30 months. The clinical and psychological characteristics of the ASD patients, the subgroup with neurodevelopmental regression (AMR) and subgroup without neurodevelopmental regression (ANMR) and the typically-developing controls are shown in Table 1. Within the ASD group, there were 20 children included in the AMR subgroup and 32 in the ANMR subgroup; two children could not be classified in these subgroups because they were adoptees, allocated by a national adoption agency. Clinical differences between these two ASD subgroups were reflected in the different behavioral tests scores obtained. All the children obtained scores above the ASD cut-off point in the ADOS-2 test. However, the patients in the AMR subgroup obtained lower scores in the Battelle developmental test than those in the ANMR subgroup, as well as higher scores in the CARS-2 test and total autism severity score in the PDBB test (Table 1).

**Table 1:** Demographic and anthropometric data in children with autism spectrum disorders (ASD) compared to controls.

	**ASD**			**CONTROL**	***P***
	**Total**	**AMR**	**ANMR**		
Age (months)	43.76 ± 11.2 43.5 (33–52.5)	43.74 ±11.9 44 (32–52.5)	43.64 ± 10.7 43 (35.5–52)	48.81 ± 18.33 51 (31–60)	ns
Gender (male)	45 (83%)	17 (85%)	27(82%)	46 (84%)	ns
Weight (kg)	16.97 ± 3.51	17.28 ± 4.17	16.65 ± 3.08	17.06 ± 4.5	ns
Height (cm)	102.7 ± 8.3	101.9 ± 8.3	102.9 ± 8.3	101.96 ± 11.1	ns
BMI (kg/m^2^)	15.94 ± 1.71	16.4 ± 1.67	15.67 ± 1.73	16.16 ± 1.74	ns
Battelle test	55.69 ± 13.8	47.05 ± 10.33	60.96 ± 13.29		0.002*
CARS test	32.6 ± 7.29	35.9 ± 8.12	30.6 ± 6.11		0.009*
PDBBI test	48.86 ± 10.82	53.93 ± 10.39	46.13 ± 10.47		0.023*

ASD, autism spectrum disorders; AMR, mental regression group; ANMR, non-mental regression group; BMI, body mass index; CARS, Childhood Autism Rating Scale Test; PDDBI, PDD Behavior Inventory. ns, no significant difference between ASD vs. control and AMR and ANMR. P* value between AMR vs. ANMR. Data are given as the mean ± standard deviation and median (interquartile range). P-values were obtained from the Mann-Whitney U-test, Student's T test, or the Fisher exact test, as appropriate. P value ASD P < 0.05 value was considered to be statistically significant.

All the proinflammatory biomarkers were detected in the plasma samples of more than 50% of the children with ASD, except for IL1β, which was detected in the plasma of only 16% of children with ASD and 6% of typically-developing children (χ2: 2,7; p: 0.141), and NGF, which was detected in 37% of children with ASD and 27% of the control children (χ2: 1,77; p: 0.183). No differences were found between the two groups in terms of the cytokine and adhesion molecule levels studied, except for NGF, in which the group of ASD children was found to have twice the plasma levels compared to the control group (Table 2).

**Table 2:** Plasma cytokine levels in patients with childhood autism (ASD) compared to the levels in the healthy control group.

**Biochemical biomarkers**	***n***	**ASD**	***n***	**Control**	***p***
C-CRP (mg/L)	54	1.9 ± 4.7	54	2.09 ± 5.09	0.77
Cathepsin D (ug/L)	54	119.5 (81.07–165.4)	54	107.2 (79.1–165.5)	0.874
IL1β (pg/ml)^a^	9	1.08 (0.6–3.07)	4	2.16 (1.2–4.24)	0.825
IL6 (pg/ml)	44	1.38 (0.34–2.98)	54	0.7 (0.24–1.46)	0.148
IL8 (pg/ml)	52	1.84 (0.84–3.82)	54	1.34 (0.67–2.77)	0.171
TNFα (pg/ml)	54	3.16 (2.2–4.56)	54	2.66 (1.83–3.69)	0.07
MPO (ug/L)	53	80.03 (54.96–148.5)	54	88.2 (61.4–141.5)	0.551
MCP−1(pg/ml)	54	122.46 (100.8–156.32)	54	117.13 (98.2–141.51)	0.228
RANTES (ug/L)	54	70.97 (41.47–135.8)	54	59.75 (28.95–94.92)	0.175
PAI (ug/L)	54	70.07 (45.52–92.65)	54	62.91 (40.62–111.53)	0.766
NCAM (ug/L)	54	392.20 ± 73.567	54	410.13 ± 72.68	0.196
ICAM (ug/L)	54	173.1 (148.85–189.9)	54	159.9 (143.2–188.6)	0.166
VCAM (ug/L)	54	1083.7 (903.8–1216.8)	54	1047.6 (840.2–1162.48)	0.283
HGF (pg/ml)	54	162.7 (136.1–222.3)	54	163.17 (131.07–194.36)	0.514
BDNF (ug/L)	54	5.04 (2.95–7.62)	54	5.36 (3.48–10.19)	0.825
NGF (pg/ml)	21	4.78 (2.93–13.18)	16	2.06 (1.25–7.36)	**0.05**

*BDNF, brain-derived neurotrophic factor; C-CRP, C-reactive protein; HGF, hepatocyte growth factor; ICAM, intercellular adhesion molecule; IL, interleukin; MCP-1, monocyte chemotactic protein; MPO, myeloperoxidase; NCAM, neuronal cell adhesion molecule; NGF, nerve growth factor; PAI, plasminogen activator factor; RANTES, regulated on activation normal t cell expressed and secreted; TNFα, tumor necrosis factor α; VCAM, vascular cell adhesion molecule*.

*Data are given as the mean ± SD or median values interquartile range. P-values were obtained from the Mann-Whitney U-test or Student's T-test, as appropriate using Bonrrefoni correction. P < 0.05 was considered to be statistically significant. P-values in bold are statistically significant*.

**Figure 1:**
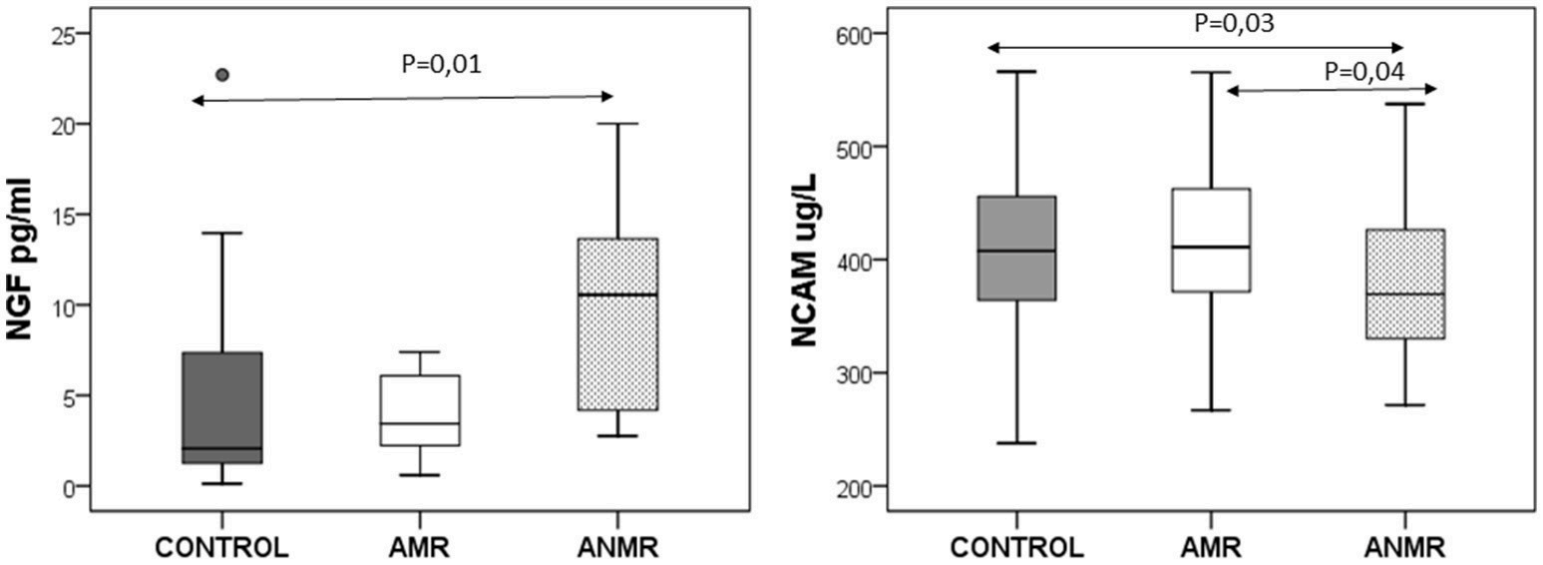
The neuronal cell adhesion molecule (NCAM) and nerve growth factor (NGF) plasma levels in children with autism subdivided as follows: AMR (mental regression group), ANMR (non-mental regression group) and a healthy control group.

Within the ASD group, the ANMR subgroup had lower NCAM plasma levels than both the AMR subgroup and typically-developing children, and higher NGF levels than the typically-developing children in the control group (Figure 1). There was no association between NCAM and NGF levels in any of the groups. There was no correlation between the levels of NCAM and NGF in either ASD subgroup, whether AMR or ANMR. No differences were identified in the levels of the molecules when comparing ASD subgroups either between high and low-functioning ASD children (in accordance with the cognitive scores from the Battelle test), or between female and male ASD children (results not shown).

Associations between plasma chemokine levels and clinical behavioral and developmental outcomes were studied. Only correlations with *r* > 0.3 are shown in Table 3. No associations between the scores obtained in behavioral and developmental tests and the cytokines in the ASD group were observed. However, within the ASD group, the ANMR subgroup exhibited a negative correlation between ICAM and language and social communication scores. In the AMR subgroup, some inflammatory parameters showed an association with repetitive behavior (MPO and RANTES), stereotypes (IL8), and social interaction scores (IL6).

**Table 3:** Significant correlations between plasma biomarker levels and behavioral impairments and associated quantitative clinical traits.

**Autism Spectrum Disorders**		**ANMR**	**AMR**		
**Tests/Biomarkers**		**ICAM**	**MPO**	**IL8**	**MCP-1**
PDDBI	EXPRESS	*r*: −0.406 *p*: 0.023			
	SOCAPP	*r*: −0.359 *p*: 0.048			
	REPRITC		*r*: 0.507 *p*: 0.038		
ADOS	Stereotypes			*r*: 0.84 *p*: 0.01	
Battelle	Personal				*r*: −0.52 *p*: 0.024
	Expression				*r*: −0.75 *p*: 0.001
	Communication				*r*: −0.71 *p*: 0.001

*AMR, mental regression group; ANMR, non-mental regression group; ICAM, intercellular adhesion molecule; MPO, myeloperoxidase; IL8, interleukin 8; MCP-1, monocyte chemotactic protein. ADOS, Autism Diagnostic Observation Schedule; CARS, Childhood Autism Rating Scale Test; PDDBI, PDD Behavior Inventory; EXPRESS, Expressive language; EXSCA, expressive social communication abilities composite; REPRITC, repetitive, ritualistic and pragmatic problems composite. SOCAPP, Social Approach Behaviors*.

## Discussion

The study results show that there are no significant differences between the levels of cytokines, cell adhesion molecules or growth factors in ASD children compared to those in typically-developing children. Nevertheless, in the ANMR subgroup of autistic children, lower plasma levels of the NCAM adhesion molecule were detected compared to the levels in the AMR subgroup and the control group. This ANMR group also exhibited higher NGF levels than the typically-developing children, which could indicate an alteration in neuronal development. These results seem to confirm the great heterogeneity of autism and the need to differentiate the pathophysiological pathways to define the phenotype spectrum and more specific and individualized therapies.

In addition to the established criteria of DMS-5 and the use of ADOS 2 for the diagnostic assessment of patients with ASD, other complementary tests were performed to evaluate the developmental level and the severity of the autistic symptomatology. These included the Battelle Developmental Inventory, PDDBI-Parent Ratings and CARS-2. These tests were instrumental in the identification of clinical characteristics in the ASD neurodevelopmental regression subgroup and ASD-diagnosed children without neurodevelopmental regression.

The immunological role in the etiopathogenesis of ASD is a matter of ongoing debate among researchers. There is evidence of an alteration in the immune system of children with ASD, which includes increased cytokine levels in both the brain and plasma (14, 26). This theory of immunological alteration is based on the knowledge that the brain is able to recognize cytokines, such as the proinflammatory cytokines IL-1a, IL-1β, TNF-α, and IL-6, as molecular signals of sickness (27). The immune system and the nervous system are in constant two-way communication, with each exerting a degree of control over the other (28).

Although many studies have reported altered levels of immune-related biomarkers or abnormal immune function in ASD, it has been surprisingly difficult to identify a consistent pattern of immunological alteration across the various studies, or develop a pathophysiological description of this alteration. For example, Onore et al. (29), have reported increased levels of proinflammatory cytokines (IL-1β, IL-6, IL-8, IL-12, p40), as well as macrophage migration inhibitory factor and platelet-derived growth factor (PDGF) in patients with autism. El-Ansary et al. (30) reported that Saudi patients with autism have markedly higher plasma HSP70, TGF-β2, Caspase-7, and INF-γ levels compared to age and gender-matched controls. Other authors have also reported that children with ASD showed altered levels of immunological parameters, including CCL2, CCL5, and CXCL9 levels, compared to healthy children (31). Recently, findings showing increased levels of S100B and TNFα in patients with ASD have been published (26). Xie et al. (25) have also observed increased blood TNFα concentrations associated with symptom severity. Indeed, the levels of TNFα in the Xie autism group showed a tendency toward significance (*p*: 0.07). It is possible therefore that the statistical significance would tend to increase if the protocol were replicated in a larger sample.

Other authors (32) by contrast, as in the present study, have found no difference between the cytokine levels of children with ASD and those of typically-developing children. For example, Napolini et al. (33) observed no increase in cytokines in a batch of up to 40 inflammatory parameters in the peripheral blood of children with ASD aged 6–8, and evidence that the immune profiles of children with ASD did not differ from those of their typically-developing siblings. Ashwood et al. (14) in an *in vitro* study and Guloksuz et al. (26) in their plasma studies also reported no differences in cytokine levels comparing different subtypes of ASD. These results cannot support a direct relation between an inflammatory status and ASD. However, an etiopathogenic implication of an immunological activation during the prenatal period has been described (34), but peripheral blood cytokine measurements may not directly correlate with changes in the central nervous system.

Cathepsin D, a protease abundantly expressed in the brain, can trigger apoptosis and secretion of cytokines, including IL-4, IL-8, IL-10, and IL-13. Malik et al. (35) have reported an increase in cathepsin D, and the corresponding increase in proinflammatory cytokines (TNFα-and IL-6) and apoptosis in autistic lymphoblasts, suggesting that cathepsin D may play an important role in cytokine-induced apoptosis. However, this study was conducted using a small sample of six children with ASD and was an *in vitro* study, which may account for the differences with the present findings.

Immune globulin-like cell adhesion molecules (CAMs) constitute a broad family, including NCAM, sICAM-1 and vCAM-1, required for tissue formation, maintenance and function. NCAM is related to a dynamic connection between cells, and decreased plasma levels of children with ASD compared to those in age-matched controls have been reported. This finding emphasizes the difference with other psychiatric pathologies, such as schizophrenia, in which these levels are elevated (36). However, other studies have found that NCAM mRNA levels were not altered in either serum samples or postmortem brain samples (37) taken from children with ASD. Jovanova-Nesic et al. (38) have also observed a decrease in the expression of NCAM-1 in the CA1 and CA3 fields of the hippocampus and, to a lesser extent, in the basal ganglia, limbic structures and cervical spinal cord.

A protective effect of glial cell line-derived neurotrophic factor (GDNF) on injured DA cells has been shown through its influence on NCAM (39). In the present study, a decrease in NCAM levels was detected in the ANMR subgroup; this may point to an incident having taken place at an earlier time, possibly during the early stages of embryonic development. The first years of development are known to be crucial for the formation of neural circuits, when there is a high predisposition to interruption. CAMs play a crucial role in many aspects of neural circuit formation and an alteration in these molecules can lead to an alteration in neuronal growth and synaptogenesis.

The plasma levels of VCAM and ICAM have been studied in high-functioning adults with ASD, revealing a decrease in the former molecule and normal levels of the latter (40). Similar results have been observed in other patients with ASD (17). However, the present study, focusing on the plasma levels of children with ASD aged 2-6, did not detect any such anomalies.

There are multiple lines of evidence indicating that immune responses and CAM levels correlate to the severity of behavioral impairments and associated symptoms and quantitative clinical traits (13, 14). Thus, dysregulation of IL-1 is implicated in impairments in memory and learning, and increases in MIP-1α, MIP-1β, and IP-10 are significantly associated with social behavior (16). Furthermore, disrupting NCAM has been shown to lead to several memory and learning deficits. In the present study, the levels of the biological parameters studied differed between the children with ASD who exhibited neurodevelopmental regression and those who did not show regression, suggesting an association between the parameters and behavioral/developmental outcomes. This finding supports the theory of different pathophysiological pathways with an earlier incident in the ANMR subgroup, as opposed to a possible subsequent aggression during the early stages of life in the AMR subgroup, something that requires clarification in future research. However, the significant association of cytokine levels with quantitative traits and the clinical subgroups analyzed suggests that altered immune responses may affect the core ASD characteristics.

An increasing body of evidence indicates that growth factors modulate motor, emotional and cognitive functions, which may explain several clinical manifestations of these disorders (41). The results regarding plasma levels of BDNF in patients with ASD (24, 42, 43) are inconsistent. BDNF, which is the most abundant neurotrophin in the CNS, can cross the blood-brain barrier, and levels in plasma and brain are highly correlated. Other growth factors have not been investigated as widely. NGF is also a member of the neurotrophin family and is involved in the growth, differentiation, survival and regeneration of nerve cells by stimulating Trk A (a transmembrane tyrosine kinase) and p75 receptors. NGF is primarily present in highly functional brain regions (41). In line with the present authors, other researchers have observed increased levels of NGF in children with ASD (44) and hyperactivity (45). In the present study, NGF was detected in fewer than 50% of the children with ASD and typically-developing children, which may be related to the early stage of life, or the sensitivity of the analysis. Second, increased NGF levels were observed in the ANMR subgroup, which also exhibited decreased levels of NCAM. Thus, although we did not observe an inverse association between these two molecules in the ANMR subgroup, the enhancement in NGF levels after brain disruption may be a part of a neuronal recovery process. This mechanism is possibly similar to the one related to NCAM and GDNF (39). HGF seems to be particularly active in the nervous system, and it plays a significant role in the neurodevelopmental process, including synapse formation (46). Significantly low levels of HGF have been described in high-functioning children with ASD (47). An association has been described between HGF serum levels and the presence of gastrointestinal disease, which is sometimes linked to ASD in subgroups of children (48). In our study, no differences were identified in the group of children with ASD.

The present observational case-control study has certain limitations. Given that the prevalence of ASD is 1%, 50 patients with ASD needed to be recruited to the study; an advantage is that clinical variability is avoided when the study is performed in a single hospital with a strict protocol and very restrictive inclusion and exclusion criteria. It is impossible to know whether the variations detected in the ASD group are a cause or a consequence of the disorder. Such highly varying results may be accounted for not only by the differences in the way this study was approached but also by the natural heterogeneity of ASD, which is still not well understood. However, this study benefits from a careful selection of children of similar ages, as well as the complete diagnosis of ASD with multiple tests, clinical follow-up and associated complementary tests. In addition, none of the ASD children were undergoing any concomitant treatment shown to modify the plasma levels of the molecules being measured. Discrepancies within the authors' results may be partly explained by the different techniques used for analysis, as the ELISA and RIAC assays are more sensitive than the Luminex assay; however, the latter allows the measurement of more analytes with a minimal volume.

In conclusion, the results of this study show that there is not a typical profile for the expression of relevant plasma cytokines, adhesion molecules or growth factors in children with ASD compared with that in typically-developing children. However, in the ANMR and AMR subgroups, some of the adhesion molecules and neuronal growth factors that could be involved in an early alteration in neurodevelopment exhibited differences. ASD is a heterogeneous pathology that converges in different ASD phenotypes; the key biomarkers in the etiology of ASD, which remains unknown, must therefore be subjected to further investigation.

## Ethics statement

This protocol was approved by the Clinical Research and Bioethics Committee at the Hospital Reina Sofia of Córdoba, respecting the fundamental principles established in the Declaration of Helsinki and informed consent was obtained from the parent or guardian of each participant before testing.

## Author contributions

AG-F, MG-C, and JP-N contributed to the study conception and study design. AG-F, KF-R, and PM-B collected all the data, acquired the behavioral data, and assisted with regulatory responsibilities. MC-R carried out the analysis. MJT-A and MG-C were responsible for the interpretation of the data, as well as drafting the manuscript. All authors read and approved the final manuscript.

### Conflict of interest statement

The authors declare that the research was conducted in the absence of any commercial or financial relationships that could be construed as a potential conflict of interest.

## Abbreviations

ASD: Autism Spectrum Disorder
AMR: mental regression group
ANMR: non-mental regression group
BDNF: brain derived neurotrophic factor
BMI: Body mass index
CARS: Childhood Autism Rating Scale Test
C-CRP, C-reactive protein HGF: hepatocyte growth factor
ICAM: intercellular adhesion molecule
IL: interleukin
MCP-1: monocyte chemotactic protein
MPO: myeloperoxidase
NCAM: neuronal cell adhesion molecules
NGF: nerve growth factor
PAI: plasminogen activator factor
PDDBI: PDD Behavior Inventory
RANTES: regulated on activation normal t cell expressed and secreted
TNFα: tumor necrosis factor α
VCAM: vascular cell adhesion molecules.
